# Heavy Metal Pollution in River Sediments: Risk Assessment, Source Apportionment, and Remediation—A Review Focusing on Chinese River Basins

**DOI:** 10.3390/toxics14090765

**Published:** 2026-08-27

**Authors:** Yuheng Tan, Jianqiao Qin, Binyi Tao, Huarong Zhao, Jinhuan Deng, Jiayin Ling, Min Dai, Xi Chen

**Affiliations:** 1 Guangdong Technology and Equipment Research Center for Soil and Water Pollution Control, School of Environmental and Chemical Engineering, Zhaoqing University, Zhaoqing 526061, China; tanyuheng003@126.com (Y.T.); kingdeng15@126.com (J.D.); daimin1007@163.com (M.D.); 2 New Energy and New Materials Research Center, Zhaoqing University, Zhaoqing 526061, China; 3 Beijing Zhongzi Huayu Environmental Protection Technology Co., Ltd., Beijing 100071, China; taobingyi@163.com; 4 College of Environmental Science and Engineering, Guilin University of Technology, Guilin 541004, China; zhaohuar@mail3.sysu.edu.cn; 5 School of Environmental Science and Engineering, Sun Yat-Sen University, Guangzhou 510275, China; chenxi90511@163.com

**Keywords:** river sediment, heavy metals, operationally defined fractionation, risk assessment, source apportionment, pollution control

## Abstract

River sediments act not only as important sinks for heavy metal pollution in watersheds, but also as potential secondary sources under changing environmental conditions. Heavy metals can enter river systems through industrial wastewater discharge, agricultural non-point runoff, urban stormwater and sewage inputs, mining and smelting activities, and atmospheric deposition. During adsorption onto suspended particles, sedimentation, and resuspension, metals such as Cd, Pb, Cr, Cu, Zn, Ni, As, and Hg progressively accumulate in sediments. Because heavy metals are persistent, non-degradable, and bioaccumulative, contaminated sediments can record historical watershed pollution while also releasing metals back into overlying water under hydrodynamic disturbance, pH and redox fluctuations, organic matter mineralization, benthic bioturbation, and dredging activities, thereby threatening aquatic ecosystem stability and human health. Using a global methodological framework with particular emphasis on Chinese river basins, this review systematically summarizes key issues in the study of heavy metal pollution in river sediments, including spatial–temporal distribution and operationally defined fractionation, pollution levels and ecological risk assessment, source apportionment, and remediation and management technologies. Current evidence indicates that heavy metal contamination in river sediments exhibits pronounced spatial heterogeneity and watershed-specific characteristics. Its distribution is jointly controlled by geological background, land use patterns, source input intensity, hydrodynamic conditions, sediment particle size composition, and organic matter content. Methodologically, the field has evolved from single total concentration monitoring and exceedance-based evaluation toward integrated assessment systems that combine total concentrations, operationally defined fractionation, bioavailability, ecological risk, health risk, and source contribution. The joint use of BCR sequential extraction, the geoaccumulation index (Igeo), the pollution load index (PLI), the potential ecological risk index (RI), the risk assessment code (RAC), sediment quality guidelines (SQGs), receptor models, isotope tracing, and machine learning has substantially improved pollution identification, risk zoning, and source apportionment. Overall, research on heavy metal pollution in river sediments has shifted from descriptive judgments of whether contamination exists toward mechanistic and management-oriented questions concerning pollution sources, risk evolution, and remediation strategies. However, important gaps remain in compound pollution transformation mechanisms, regional background values and evaluation benchmarks, uncertainty in model parameters, long-term dynamic monitoring, and engineering-scale verification of remediation technologies. Future studies should strengthen multi-media, multi-scale, and long-term monitoring and further integrate fractionation analysis, toxicological effects, source apportionment models, and remediation technologies to provide a scientific basis for watershed ecological security and precision management of contaminated sediments.

## 1. Introduction

### 1.1. Research Background and Significance

#### 1.1.1. Global Severity of Heavy Metal Pollution in Aquatic Environments

Heavy metal pollution in aquatic environments is a major challenge for global water ecological security. Continuous inputs from industrial wastewater, agricultural runoff, municipal sewage, and atmospheric deposition have led to the long-term accumulation of Cd, Cr, Cu, Ni, Mn, Fe, and other elements in diverse water bodies. Although Pb and Zn concentrations have declined in some regions owing to policy interventions, the overall pressure of combined contamination remains substantial [1,2,3,4,5,6,7,8,9].

Sediments are key media controlling heavy metal migration and transformation. Their pollutant loads are often higher than those of overlying water, and they can cause long-term ecological toxicity and health risks through resuspension, desorption, and food chain enrichment. Studies of coastal zones, lakes, and urban rivers show that sediment contamination is commonly controlled by both geological background and anthropogenic inputs such as agriculture, navigation, and industry [10].

In developing countries, mining, smelting, chemical manufacturing, and intensive agriculture make compound heavy metal pollution in sediments particularly prominent. In urban rivers of China’s nine major river systems, Pb, Cu, Zn, Cd, Cr, Ni, As, and Hg are widely accumulated and often coexist with emerging pollutants such as microplastics, increasing the complexity of risk identification and remediation [11,12,13,14,15,16,17,18,19,20].

In summary, from the persistent deterioration of global aquatic environments to overlapping contamination in coastal sediments and source pressures across China’s large river systems, aquatic heavy metal pollution shows extensive geographical coverage, complex toxicity mechanisms, and long-term concealment. These features highlight the need for watershed-scale frameworks that integrate precise source tracing, ecological assessment, and macro-scale management.

#### 1.1.2. Pollution Status of Key River Basins in China

Key river basins, lakes, and reservoirs in China are affected by historical discharge, urbanization, agricultural intensification, and hydrological engineering. As a result, heavy metal pollution in sediments is characterized by strong spatial heterogeneity, mixed sources, and transport processes significantly modified by engineering regulation [11,21].

In southern China, the Yangtze River system and surrounding lakes and reservoirs are mainly influenced by mining, tributary inputs, and water conservancy projects. Studies in Poyang Lake and the Xiangjiang River indicate that lake inlets, downstream mining areas, and low-flow zones tend to accumulate As, Cu, Zn, Pb, and Cr, whereas regions with higher vegetation cover may show partial attenuation [22,23,24,25].

In the Yellow River basin of northern China, heavy metal pollution reflects the combined influence of irrigation in arid regions, traffic emissions, natural weathering, and water-sediment regulation. In the Ningxia section, Mn, As, Ni, Cr, and Zn exceed background values, and water-sediment regulation can alter metal transport fluxes through resuspension and sediment conveyance [26,27,28,29,30].

High ecological risks also occur in densely populated basins in northeastern and eastern China. Cd risk is prominent in the middle and lower Songhua River, whereas enclosed or semi-enclosed systems such as the Tongyang River in the Chaohu Lake basin remain affected by agricultural fertilization, industrial relocation legacies, and runoff inputs [31,32,33,34].

Overall, heavy metal pollution in key Chinese basins is shaped by a complex mixture of mining and smelting, agricultural non-point sources, traffic emissions, and natural weathering, and is further reshaped by dams and water-sediment regulation. This complex source–transport–sink dynamic emphasizes the need for spatially explicit source apportionment and cross-media risk assessment at the watershed scale [35,36].

#### 1.1.3. Source-Sink Mechanisms of Sediments in Aquatic Ecosystems

Sediments retain heavy metals through adsorption, precipitation, flocculation, and particle consolidation, serving as both historical archives and major sinks of watershed pollution. When external inputs are strong or fine-particle deposition is significant, this accumulation can amplify internal pollution risks [2,21,37,38].

However, the sink function of sediments is not stable. Increased temperature, decreased dissolved oxygen, pH and Eh fluctuations, and hydrodynamic disturbance can promote desorption, speciation transformation, and resuspension, converting sediments from pollution sinks into secondary sources for overlying water [39,40].

Biological processes are integral to sediment source–sink behavior. Bioturbation and bioirrigation by benthic organisms can resuspend fine particles and modify oxygen penetration, pH, and redox microgradients. Microbial metabolism can mediate metal complexation, redox transformation, methylation, and remobilization, whereas uptake by benthic fauna and subsequent trophic transfer link sediment-bound fractions to ecological exposure. Biological community structure and activity should therefore be evaluated together with physicochemical and hydrodynamic controls when estimating sediment stability and secondary-release risk [13,41,42,43].

Changes in hydrology and hydrodynamics are important external triggers for source–sink conversion. High-intensity processes such as water-sediment regulation in the Yellow River can disrupt bed stability, transfer particle-bound metals into the suspended phase, and redistribute contaminants downstream, thereby changing exposure risks [27,28,44].

Accordingly, source–sink behavior in river sediments is not a one-way sedimentation process but a complex ecological cycle governed by physicochemical evolution, biological mediation, and hydrodynamic engineering disturbance. Quantifying thresholds and release potentials for sink-to-source conversion is essential for cross-media ecological risk assessment and for developing in situ stabilization technologies to interrupt internal loading [41].

### 1.2. Research Evolution and Development Trends

#### 1.2.1. Early Stage: From Single-Concentration Monitoring to Total-Load Control

Early studies mainly relied on total concentration monitoring, comparing measured values with background values or quality standards to classify pollution levels. Indices such as PLI and RI provided basic tools for pollution identification, but this stage paid limited attention to speciation, bioavailability, and migration processes [2,18,45].

The concept of total-load control further emphasized watershed-scale pollution-load accounting and cross-media transport fluxes, shifting research from local concentration description toward integrated assessment of annual inputs and outputs, spatial accumulation, and hydrodynamic transport [22,33,35].

#### 1.2.2. Intermediate Development: Introduction of Sequential Extraction Fractionation (BCR) and Ecological Risk Assessment

Intermediate-stage research recognized that total concentration is not equivalent to toxicity. In this review, chemical speciation denotes distinct molecular, complexation, or oxidation state forms, whereas Tessier and BCR sequential extraction yield operationally defined fractions determined by prescribed reagents and conditions. BCR separates acid-soluble, reducible, oxidizable, and residual fractions and is used here as an indicator of relative mobility and potential release, not as direct identification of discrete chemical species [1,21,22].

Sequential extraction fractionation shifted risk assessment from simple concentration levels to release potential and bioavailability. Acid-soluble and exchangeable fractions are sensitive to pH and Eh changes, and higher non-residual proportions of Cd and Zn often indicate greater ecological sensitivity. RAC evaluates risk levels using the proportion of readily available fractions [34,46,47].

Ecological risk assessment also evolved from single-index evaluation toward multi-index integration. SQGs, TEL/PEL, RI, and RAC reflect toxicity thresholds, potential ecological risk, and fractionation-based availability, respectively; multi-model integration is now widely used to improve reliability [2,45,46].

#### 1.2.3. Current Frontier: Refined Source Apportionment Based on Receptor Models and Machine Learning

Current research increasingly focuses on refined quantitative analysis of source contributions. Receptor models such as PMF, APCS-MLR, and UNMIX can identify potential sources and estimate contribution rates without complete source profiles, promoting a shift from pollution description to causal diagnosis and zone-specific management [9,39,48,49].

To reduce uncertainty in identifying sources with similar geochemical signatures, isotope tracing has been coupled with receptor models. Stable isotopes of Cd, Pb, and Zn provide source fingerprints that can distinguish smelting, fertilization, fossil fuel combustion, and natural weathering, and can validate PMF model results [46,50,51].

Machine learning provides rapid diagnostic tools for high-dimensional and nonlinear environmental data. Random forests, support vector machines, and related algorithms can integrate environmental magnetism, spatial factors, and pollutant concentrations to support pollution identification, risk prediction, and validation of source apportionment results, forming an intelligent early diagnosis warning monitoring pathway [9].

### 1.3. Literature Search Strategy and Study Selection

A structured literature search was conducted from 1 April to 20 May 2026 and updated on 25 May 2026. Sources comprised the Web of Science Core Collection, SpringerLink, and the China National Knowledge Infrastructure (CNKI), which yielded 198, 30, and 80 records, respectively. Ten additional records were identified through backward citation tracking and supplementary full-text retrieval. The search covered literature available up to 25 May 2026 without a lower publication year limit.

The search combined three concept blocks: (1) sediment setting (“river sediment*” OR “stream sediment*” OR “fluvial sediment*” OR “reservoir sediment*” OR “estuarine sediment*” OR “dredged sediment*”); (2) contaminants (“heavy metal*” OR “potentially toxic element*” OR cadmium OR Cd OR lead OR Pb OR chromium OR Cr OR copper OR Cu OR zinc OR Zn OR nickel OR Ni OR arsenic OR As OR mercury OR Hg); and (3) outcomes and methods (“risk assessment” OR “ecological risk” OR “source apportionment” OR fractionation OR BCR OR Igeo OR PLI OR RI OR RAC OR SQG* OR PCA OR PMF OR UNMIX OR isotope* OR “machine learning” OR remediation). Terms within a block were joined by OR and blocks by AND; equivalent Chinese terms were used in CNKI.

Eligible records examined river, stream, estuarine, reservoir, or dredged sediments; addressed at least one heavy metal or potentially toxic element; reported distribution, operationally defined fractionation or bioavailability, pollution or ecological risk, source apportionment, or remediation/management; and provided sufficient full text. English- and Chinese-language journal articles, reviews, dissertations or theses, and selected authoritative books or official reports were eligible. Conference abstracts, duplicates, non-sediment studies, records without substantive heavy metal analysis, and records without relevant assessment, source, or management information were excluded.

**Figure 1:**
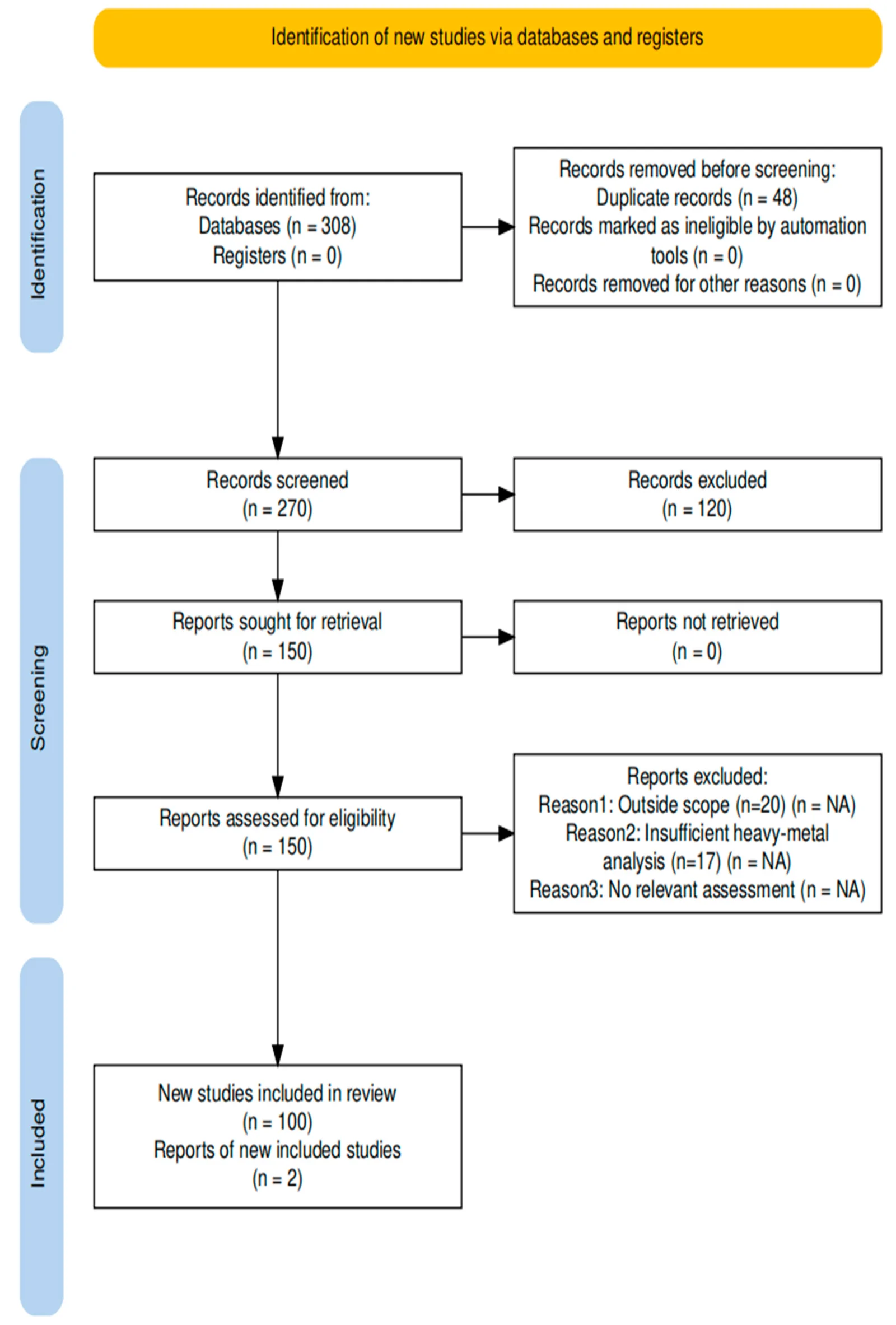
PRISMA 2020 flow diagram for literature identification, screening, eligibility assessment, and inclusion. Counts were retrospectively reconstructed from the reported database totals and final literature library.

**Figure 2:**
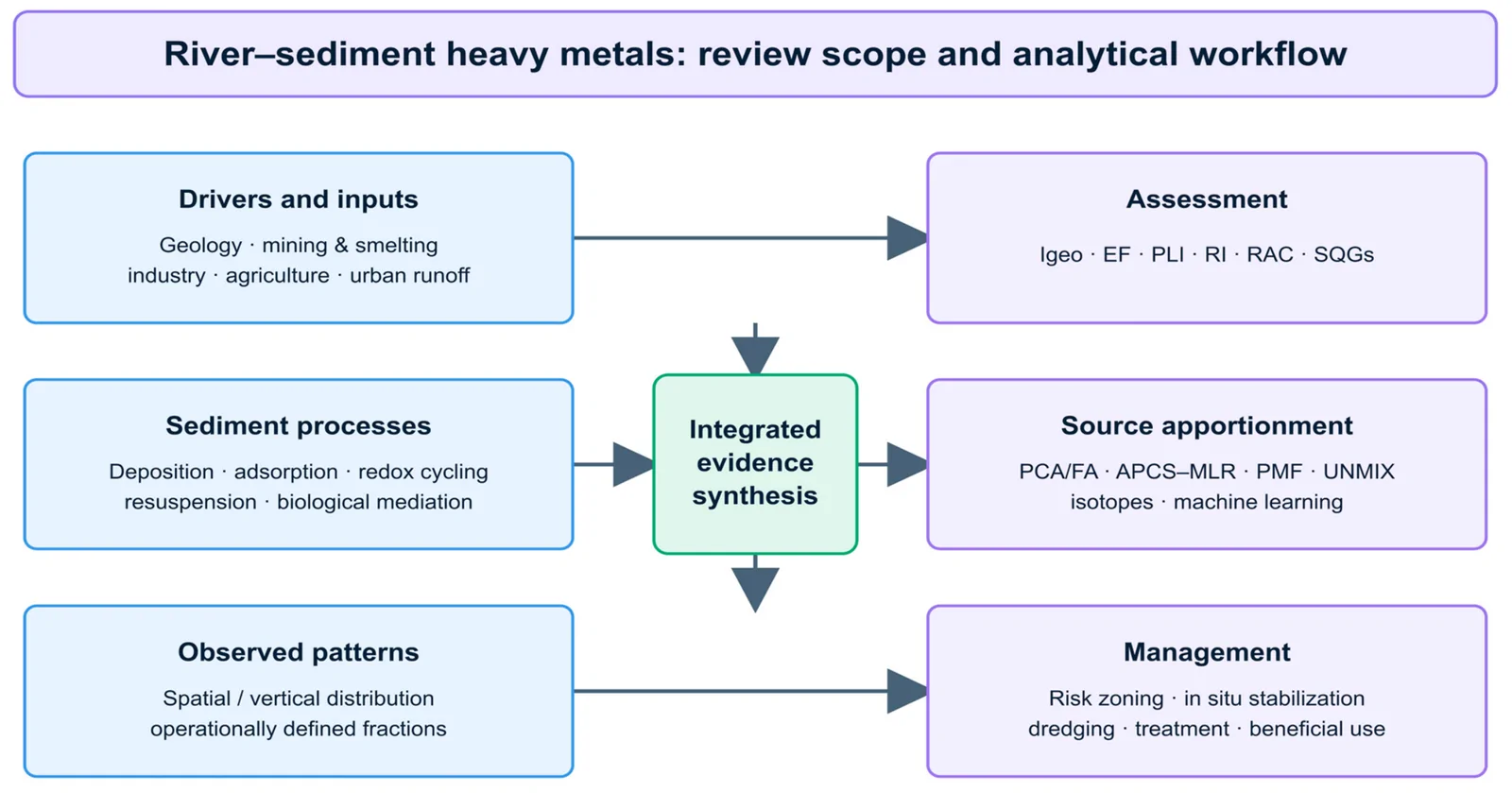
Scope, evidence synthesis, and analytical workflow for the review of heavy metal pollution in river sediments [52,53,54]. Arrows indicate the principal analytical links among drivers and inputs, sediment processes, observed patterns, integrated evidence synthesis, assessment, source apportionment, and management.

Records were manually deduplicated using titles, authors, publication years, DOIs, and study locations. Title/abstract screening was followed by full-text assessment; all reports selected for full-text review were retrieved. The reconstructed PRISMA record identified 318 records, removed 48 duplicates, screened 270 titles/abstracts, excluded 120, assessed 150 full texts, and excluded 50 full texts (outside scope, *n* = 20; insufficient heavy metal analysis, *n* = 17; no relevant assessment/source/management information, *n* = 13). The original review therefore included 100 sources. During revision, the two studies recommended by the reviewers were assessed and added, producing a final evidence base and reference list of 102 sources. Because the original search strings and screening log were not saved prospectively, the search strings and stage counts were reconstructed from the author’s database totals and final reference library during revision. The study-selection process is summarized in Figure 1, and the overall review scope, evidence synthesis, and analytical workflow are shown in Figure 2.

## 2. Spatial–Temporal Distribution and Operationally Defined Fractionation of Heavy Metals in River Sediments

### 2.1. Macro- and Micro-Scale Spatial Distribution Patterns

#### 2.1.1. Spatial Heterogeneity at the Watershed Scale

At the watershed scale, heavy metals in river sediments exhibit pronounced spatial heterogeneity controlled by upstream inputs, urbanization, point-source discharge, land use, and hydrodynamic conditions. Downstream reaches, industrial zones, tributary confluences, and low-flow depositional areas are more prone to metal enrichment [12,18,39,52,53,55,56,57].

Reservoirs, dams, river–lake confluences, and estuarine salinity mixing alter particle deposition and resuspension, making river–reservoir transition zones, fore-dam areas, and estuarine depositional regions important pollutant accumulation hotspots. This indicates that spatial distribution is controlled by source–transport–sink linkages rather than uniform diffusion [58,59,60].

Modern assessment systems based on spatial geostatistics, including geographically weighted regression, further demonstrate that heavy metal deposition in both dammed inland freshwater networks and tide-influenced estuarine zones is strongly governed by macroscopic flow gradients and terrigenous input fluxes [61]. Therefore, accurate characterization of source–transport–sink cycling is central to understanding spatial distribution at the watershed scale.

#### 2.1.2. Vertical Profile Characteristics

Vertical sediment profiles record historical pollution inputs and environmental remediation processes. Surface sediments are more strongly influenced by current emissions and water exchange, whereas deeper layers more often reflect historical deposition or geological background. Core samples are therefore useful for reconstructing accumulation, burial, and release potential [21,62].

Vertical migration is also controlled by speciation, particle size, organic matter, salinity, and redox conditions. Cd and Cu with high acid-extractable or exchangeable fractions can diffuse more readily under environmental disturbance, while high salinity or strong hydrodynamics can further modify sediment–water partitioning [22,63,64].

Dredging, wetland restoration, and river regulation can remove or cover highly contaminated surface layers, causing discontinuities in vertical pollution records. Interpretation of vertical profiles should therefore consider natural sedimentation, historical pollution inputs, and human remediation events [65,66,67,68].

#### 2.1.3. Identification of Typical Exceeding Heavy Metals

Identifying elements that exceed standards is fundamental for pollution assessment and risk management. Most watershed studies focus on Cd, Pb, Cu, Zn, Cr, Ni, As, and Hg, among which Cd is frequently a dominant ecological risk factor because of its high toxicity coefficient and bioavailability [1,9,18].

Element spectra vary spatially according to differences in human activity types and intensities. Geoaccumulation index (Igeo) results indicate that Ni, Cr, Pb, Cu, Zn, and Cd are generally within the range from uncontaminated to moderately contaminated, whereas Hg enrichment in some areas makes it a priority element for management [39,69,70].

Relationships among elements with shared geochemical affinity provide a useful complementary line of evidence. In the Matipó River basin, a tributary of the Doce River in Minas Gerais, Brazil, Reis et al. compared spatial co-variation among Cu–Zn, Pb–Cu–Zn, and Cr–Ni with Goldschmidt affinity classes, local lithology, and land use. Similar behavior among affiliated elements supported a lithogenic influence at some sites, whereas divergence from the local geochemical pattern and weak correlations helped identify sites affected by agriculture or other human activities. Such relationships should be calibrated against local parent material, mineralogy, grain size, and redox-sensitive Fe/Mn phases and interpreted together with enrichment indices and multivariate models rather than used as fixed universal ratios [71].

Among typical exceeding elements, Cd and Hg require particular attention because of their strong biotoxicity, environmental persistence, and potential for biomagnification through aquatic food webs [29,40,64,68].

### 2.2. Effects of Special Hydrological Systems on Heavy Metal Distribution

#### 2.2.1. Retention Effects of Cascade Reservoirs and Dams

Large-scale water conservancy projects fundamentally reshape natural river hydrological rhythms and hydrodynamic characteristics. Cascade reservoirs and dams therefore exert marked retention effects on the migration and transformation of sediments and associated heavy metals [27]. During the transition from natural rivers to river–reservoir systems, physical dam barriers and reservoir regulation sharply reduce flow velocity and sediment-carrying capacity in impounded and fore-dam areas.

Field investigations across regions confirm this retention-driven enrichment. Cascade reservoirs enhance the accumulation of heavy metals in surface sediments and may intensify local micro-ecological risks [9,22]. In urban river–reservoir systems such as the Feiyun River and Dongtiaoxi River basins, reservoir wetlands and surface sediments continuously receive trapped upstream materials and become core zones of multi-phase heavy metal accumulation, with sediment pollutant accumulation rates far exceeding natural background levels [39,60,72,73].

Nevertheless, the retention effect does not simply reduce downstream pollution loads; it may also store long-term ecological risks within reservoir sediments. Flood discharge, scouring, pH decline, redox changes, or bottom-water hypoxia can desorb accumulated metals and reintroduce them into overlying water, causing internal secondary pollution. Reservoir sediments should therefore be prioritized in watershed pollution prevention and risk zoning [9,40].

#### 2.2.2. Enrichment Mechanisms in River–Lake Confluences and Estuaries

River–lake confluences and estuaries are not only physical boundaries between water bodies but also important biogeochemical hotspots in aquatic ecosystems [58,74]. Owing to sharp hydrodynamic transitions and physicochemical gradients, these transition zones readily become natural sinks for persistent pollutants such as heavy metals.

First, abrupt hydrodynamic changes are a dominant mechanism driving spatial metal enrichment [44]. In Poyang Lake, for example, flow direction promotes pollutant convergence toward downstream and river–lake confluence areas, while differential settling of particles with different sizes produces clear concentration gradients.

Second, increased salinity and electrical conductivity in estuaries and river–salt lake transition zones compress the electrical double layer on suspended particles, weaken electrostatic repulsion, and promote flocculation and deposition of fine particles and colloids. Because many heavy metals are transported by suspended particles, this process enhances metal enrichment in transition zone sediments [74,75].

However, confluences and estuaries are not static terminal repositories. Complex salinity gradients and periodic hydrological rhythms create high uncertainty in source-sink conversion. Dissolved alkali and alkaline-earth cations such as Na^+^, K^+^, and Ca^2+^ in high-salinity water can compete with heavy metal ions for adsorption sites, displacing previously adsorbed metals and causing their release and resuspension, thereby inducing secondary risks.

### 2.3. Operationally Defined Fractionation and Bioavailability of Heavy Metals in Sediments

#### 2.3.1. Sequential Extraction Methods for Operationally Defined Fractionation

A basic consensus in sediment heavy metal assessment is that total concentration alone cannot fully represent ecological threat. Molecular-level chemical speciation and operationally defined fractionation are related but not equivalent. Sequential extraction estimates fractions released under prescribed reagents and conditions; these fractions are commonly interpreted as proxies for relative mobility and potential bioavailability, but they should not be treated as uniquely identified chemical species [21,22].

Sequential extraction procedures are widely used to quantify metal speciation, particularly the Tessier five-step method and the BCR three-step procedure. Acid-soluble fractions are weakly bound and can be released under slight environmental changes such as pH decline, showing high bioavailability; reducible fractions are sensitive to Eh changes and can be released under anoxic conditions [47].

In addition to classical sequential extraction, habitat-specific methods continue to develop. The relationship between acid volatile sulfide (AVS) and simultaneously extracted metals (SEM) is commonly used to evaluate potential toxicity in anoxic sediments. When the SEM/AVS ratio exceeds a threshold, metal bioavailability increases significantly. In industrial urban rivers and coastal flats, sewage, exhaust emissions, and industrial wastewater can lead to sustained accumulation of highly reactive metals [9,18,47,69].

In summary, operationally defined fractionation provides an important basis for environmental quality assessment and for understanding metal migration and transformation at sediment–water–biota interfaces. Future studies should combine fractionation data with bioaccumulation models and health risk indicators to characterize dynamic food chain exposure and support in situ remediation of contaminated sediments.

#### 2.3.2. Operationally Defined Fraction Patterns of Typical Heavy Metals

The ecological effects of heavy metals in sediments depend not only on total concentrations but also on operationally defined fraction distribution. Different metals show distinct distributions among acid-soluble, reducible, oxidizable, and residual fractions, controlled by geochemical properties, source inputs, and sediment–water interface conditions.

For highly toxic and bioaccumulative metals such as Cd and Pb, existing studies generally show high proportions of non-residual fractions, especially acid-soluble and reducible fractions. These active fractions indicate strong mobility and direct bioavailability. Cd is highly sensitive to minor changes in aquatic conditions and can be readily desorbed and secondarily released, whereas Pb is often enriched in the reducible fraction and strongly associated with amorphous Fe-Mn oxides, creating a high reactivation risk under hypoxia or rapid redox shifts.

By contrast, Cu and Zn fractionation patterns reflect strong complexation with organic matter and sulfides. Hydrochemical studies in industrial urban rivers and shallow lakes show that Cu readily forms stable complexes with natural organic matter; therefore, in sediments strongly disturbed by urban sewage and industrial wastewater, Cu commonly dominates the oxidizable fraction associated with organic matter and sulfides [47,69,76].

Cr and As fractionation has also received increasing attention in coastal flats and pollutant-receiving basins [45,74]. Intensified industrial activities and atmospheric deposition increase the proportion of highly active toxic fractions, such as acid-soluble Cr and As, in surface sediments [47]. This shift from stable residual forms toward active non-residual forms facilitates uptake by benthic organisms and submerged plants in coastal transition zones [42,74,77].

#### 2.3.3. Environmental Controls on Redistribution Among Operationally Defined Fractions

Heavy metal distribution among operationally defined fractions in sediments is not isolated or static but evolves dynamically under non-equilibrium geochemical conditions at the overlying water–sediment interface [27,39]. Such redistribution responds directly to changes in environmental conditions and determines secondary release potential and bioavailability [5,22].

Redox potential (Eh) and dissolved oxygen (DO) fluctuations in bottom water are key drivers of metal transfer across the interface. Under reducing conditions, Cd, Cu, Zn, and Pb can form relatively stable metal sulfide precipitates. However, oxygenated water intrusion caused by benthic disturbance, dry season exposure, or dredging can oxidize and decompose metal sulfides, releasing heavy metals [22,78,79].

pH changes and organic matter mineralization can also trigger speciation transformation. Acidification generally enhances metal desorption and migration. Weak alkaline conditions may promote precipitation, but can also stimulate dissolved organic carbon (DOC) release and form mobile organic–metal complexes with Cu and Pb.

Physical hydrological disturbance and climate change are increasingly recognized as catalytic factors. Seasonal flow pulses, flood scouring, and wind-wave-induced increases in bed shear stress can cause large-scale sediment resuspension [44]. This process rapidly disrupts sediment closure, intensifies gas–water–sediment exchange, and releases free and weakly bound metals into the water column, generating severe secondary ecological exposure risks [40,44].

Overall, heavy metal fraction distribution in sediments is dynamically regulated by pH, Eh, hydrodynamics, and climate conditions. Clarifying these response mechanisms helps identify secondary pollution risks and provides a basis for in situ stabilization, capping, and habitat regulation.

## 3. Pollution Levels and Risk Assessment Methods for Sediment Heavy Metals

### 3.1. Qualitative and Semi-Quantitative Models for Pollution Assessment

The development of qualitative and semi-quantitative models for pollution assessment has so far mainly included the following types. The main stages, methods, outputs, and implications are summarized in Table 1.

#### 3.1.1. Geoaccumulation Index (Igeo)

The geoaccumulation index (Igeo) is a classical model for assessing heavy metal pollution and is commonly used to evaluate element enrichment in sediments or soils relative to background values. By introducing a background correction factor, this method partially reduces the influence of natural geological differences.

Igeo = log_2_ [Cn/(k × Bn)]

where Igeo is the geoaccumulation index; Cn is the measured concentration of the nth heavy metal element (mg/kg); k is a correction coefficient for lithogenic background variation, typically set at 1.5, and is used to minimize background fluctuations caused by lithological variation or natural weathering [80]; and Bn is the natural background value of the corresponding element.

In practice, the selection of the regional background value (Bn) is a major source of variation in Igeo results. Different studies choose background values according to watershed characteristics and research objectives. For example, in source-oriented risk assessment of the Yixun River basin, researchers used the soil background value of Hebei Province as the reference standard [77].

With appropriate background values, Igeo can reveal spatial gradients of metal enrichment. Some river studies show that Cu and Cd remain close to background levels upstream, whereas Igeo values increase to slight or moderate contamination downstream or midstream, reflecting enhanced anthropogenic inputs along the river course [81].

#### 3.1.2. Application of the Pollution Load Index (PLI) and Enrichment Factor (EF)

Enrichment factor (EF) and pollution load index (PLI) are widely used in the assessment of heavy metal pollution in aquatic sediments. They emphasize different dimensions of assessment and interpretation, and their combined use can improve systematicity and credibility.

Özbay [10] jointly applied EF, PLI, Igeo, and contamination factors to coastal surface sediments in Mersin Bay, northeastern Mediterranean. EF used Fe as a reference element for normalization, thereby reducing the effects of grain size differences and geochemical background and helping distinguish natural weathering from anthropogenic inputs for Cr, Ni, and Cd.

Talukder et al. [82] used Al as the normalizing element to calculate EF in Setiu Wetland sediments, reducing the influence of grain size and mineralogical composition. Their results indicated clear anthropogenic enrichment of Cr, whereas other metals were mainly of natural origin. Thus, EF is suitable for identifying single-element enrichment sources, while PLI is more appropriate for classifying overall pollution.

Studies in the Ganges River basin [83,84] combined background correction, concentration normalization, and source identification, and used PLI to evaluate multi-metal pollution. Significant integrated pollution was identified in reaches such as Prayagraj and Banshberia, further demonstrating the applicability of PLI for large-scale watershed comparisons [84].

#### 3.1.3. Application of Sediment Quality Guidelines (SQGs) in Toxicity Prediction

In aquatic ecosystems, heavy metals can be transferred through food webs, making ecological risk and toxicity assessment of sediment-bound metals highly important [46]. Actual metal toxicity depends not only on total concentration but also on mobility and bioavailability, especially labile fractions [46]. Therefore, toxicity prediction based on SQGs should combine total concentration assessment with available fraction analysis.

The Nanfei River example below is retained as an application from the China-focused evidence base rather than presented as a globally representative benchmark. Because SQG derivation, sediment properties, contaminant mixtures, and receptor sensitivity vary among jurisdictions, numerical thresholds should be applied only after confirming their regulatory provenance and regional suitability.

In a case study of surface sediments from the Nanfei River, an urban river, SQGs were used to predict and classify ecological toxicity risks of heavy metals. The results showed that heavy metals in Nanfei River sediments generally presented low to moderate risks according to inherent SQG thresholds [46].

SQGs were particularly useful for identifying extremely high ecotoxicity. In this case, the SQG assessment captured regional high-risk sites: Pb at one sampling site and Zn at three sampling sites exceeded the probable effect concentration (PEC), suggesting very high ecological toxicity risk if left uncontrolled [46].

To further validate and supplement SQG toxicity prediction, diffusive gradients in thin films (DGT) were introduced to analyze labile metal concentrations. The average DGT-measured concentrations were Cu (4.08 μg/L), Cd (0.21 μg/L), Zn (54.63 μg/L), Pb (0.17 μg/L), As (0.20 μg/L), Cr (1.74 μg/L), and Hg (2.69 μg/L) [46].

#### 3.1.4. Potential Ecological Risk Index (RI)

The potential ecological risk index (RI), proposed by Hakanson, is a classical and widely applied method for evaluating heavy metal risk in aquatic sediments. It integrates metal enrichment, environmental sensitivity, and biological toxicity through single-element risk factors and an integrated risk index.

RI = ΣTir × Cir

where Tir is the toxic-response factor for an individual contaminant, and Cir is the contamination factor.

This method comprehensively considers pollutant concentration, toxicity level, and receptor sensitivity to risk sources.

Bensadi and Azzoug [85] applied the traditional RI method to nine elements (Mn, Fe, Cr, Ni, Cu, Zn, As, Cd, and Pb) in sediments of the Soummam River, Algeria. Cd showed single-element risk factors of 193–955.5, reaching high to very high risk and becoming the dominant risk driver.

Siddique et al. [86] assessed heavy metal contamination in surficial sediments from the Lower Meghna River Estuary on the Noakhali coast of Bangladesh. This estuarine case underscores the need to interpret enrichment and ecological risk indices against local sediment texture, mineralogy, and coastal hydrodynamics.

Bai et al. [87] optimized toxic-response coefficients according to regional metal characteristics in sediments of the Yu River, a polymetallic ore concentration area, and used RI for spatiotemporal comparison across hydrological periods. Their results showed that Mo, W, Cu, Pb, and Zn mining activities produced higher ecological risk upstream than downstream, with clear variation among flood, dry, and normal-flow periods.

Zhu et al. [60] combined RI with spatial distribution analysis in surface sediments of the Feiyun River river–reservoir system. Cd, Zn, and As were significantly enriched in reservoir and fore-dam areas under cascade reservoir retention and sediment deposition, and integrated ecological risk decreased from upstream to downstream.

In summary, RI can rapidly identify key elements and high-risk areas and is applicable to rivers, mining areas, and river–reservoir systems. However, it mainly relies on total metal concentrations and does not incorporate speciation or bioavailability; background values and toxic-response coefficients may also be regionally subjective. In complex geological or highly disturbed areas, Igeo, EF, RAC, and other methods should be combined to improve accuracy and reliability [6,7,52,53].

## 4. Technical System for Source Apportionment of Heavy Metals in Sediments

### 4.1. Classification and Evolution of Source Apportionment Techniques

#### Concept and Classification of Source Apportionment

Source apportionment refers to the use of physical, chemical, and mathematical methods to analyze pollutants in environmental receptors such as air, water, and soil, identify emission sources qualitatively, and quantify the contribution of each source to environmental pollution. Principal component analysis (PCA) and cluster analysis (CA) are widely used for qualitative source identification.

### 4.2. Multivariate Statistical Methods

#### 4.2.1. Role of HCA and Pearson/Spearman Correlation Analysis in Identifying Mixed Sources

Cluster analysis (CA) is an exploratory data analysis method based on similarity measures. It groups heavy metals according to inter-element associations and thereby helps identify potential sources. A study in the eastern valley of Mosul City similarly indicated that urban runoff and industrial discharge were important potential sources, causing significant spatial variation in heavy metal concentrations [51].

Although CA does not require prior knowledge, interpretation of clusters must rely on professional understanding of sediment physicochemical interactions. Mechanistically, adsorption and desorption kinetics between heavy metals and sediment particles are strongly affected by pH, organic matter content, and sediment texture [51].

At broader scales, preliminary analyses based on clustering and correlation ultimately support risk warning and management. When sediment metals show high biological toxicity risk—for example, when mPEC-Q values fall between 1 and 5 [9]—source identification becomes particularly urgent. However, in sensitive regions such as polymetallic mining areas, the lack of systematic monitoring networks poses substantial challenges for early warning and later forecasting [88].

#### 4.2.2. Principles and Limitations of PCA/FA for Extracting Source Fingerprints

Principal component analysis (PCA) is a core statistical method for identifying potential sources of sediment heavy metals. It reduces high-dimensional data by using inter-variable correlations and extracts principal factors with similar geochemical behavior. Before PCA is applied to source fingerprint extraction, data suitability is usually tested. Based on strong correlations, PCA can extract major components; for example, PC1 with an eigenvalue of 3.948 and a variance contribution of 43.869% showed high positive loadings for Fe, Cr, Pb, and Cd, indicating a common source.

PCA and common factor analysis (FA) are related but distinct. PCA decomposes total observed variance into orthogonal components and is mainly a dimension reduction method. FA models shared covariance through latent factors and separates common variance from variable-specific and error variance. Rotated loadings may suggest source groupings, but factor retention, rotation, scaling, sample adequacy, and independent environmental evidence must be reported before a statistical factor is assigned to a physical source.

In complex watershed habitats, receptor models can effectively decompose mixed pollution features. In a river–reservoir system (MLHR), source identification classified metals causing high biological toxicity risk (mPEC-Q values of 1–5) into categories such as traffic, agriculture, and partial industrial mixed sources containing Pb, Hg, Zn, Cu, and As. Receptor models such as UNMIX have also been widely applied to improve source fingerprint identification [51,53].

However, PCA/FA and related statistical models cannot by themselves identify physical sources. Results are sensitive to scaling, collinearity, outliers, rotation choices, and spatial dependence, and these models do not explicitly represent contaminant transport. Interpretations should therefore be supported by land use, spatial patterns, source profiles, isotope evidence, receptor models, or other independent process-based information.

#### 4.2.3. Machine Learning Approaches for Source Apportionment and Validation

Machine learning methods such as random forests and support vector machines can model nonlinear relationships among metal concentrations, sediment properties, land use, and spatial variables. They can support pollution classification, variable screening, source pattern recognition, and spatial prediction where linear assumptions are inadequate [9].

Advantages include flexible nonlinear fitting and accommodation of high-dimensional predictors. Limitations include overfitting, small or imbalanced training sets, spatial autocorrelation, limited interpretability, and poor transferability among watersheds. Robust studies should report preprocessing and hyperparameter selection and use nested or spatial cross-validation, independent testing where possible, sensitivity analysis, and comparison with receptor model, isotope, or source-profile evidence. Machine learning should complement rather than replace process-based interpretation.

As a cross-contaminant methodological comparison, Trajković et al. investigated 16 PAHs in 25 sediment samples from Bubanj Lake, an urban shallow lake in Kragujevac, Serbia, using multivariate statistics, diagnostic ratios, PMF, and Monte Carlo probabilistic health risk analysis [89]. Although PAHs differ from heavy metals in sources and environmental behavior, the study demonstrates transparent integration of receptor modelling and uncertainty analysis.

### 4.3. Typical Receptor Models

#### 4.3.1. Application Principle of the APCS-MLR Model

The absolute principal component scores-multiple linear regression (APCS-MLR) model converts PCA factor scores into absolute principal component scores and then uses multiple linear regression to calculate source contribution rates. The model can qualitatively identify metal loadings on pollution sources while quantitatively determining contribution rates [54].

#### 4.3.2. Positive Matrix Factorization (PMF)

Positive matrix factorization (PMF), recommended by the US Environmental Protection Agency as a source apportionment tool, was proposed by Paatero and Tapper in 1993 as a constrained weighted least-squares method.

In the model, X_ij_ is the concentration of element j in sample i; g_ik_ is the contribution concentration of source k to sample i; fkj is the mass fraction of element j in source k; and e_ij_ is the residual.

PMF decomposes the concentration matrix as x_ij_ = Σ_k=1_^p^ g_ik_f_kj_ + e_ij_, where x_ij_ is the concentration of species j in sample i, g_ik_ is the contribution of factor k to sample i, f_kj_ is the profile of species j in factor k, and e_ij_ is the residual [89].

The weighted objective function is Q = Σ_i=1_^n^Σ_j=1_^m^(e_ij_/u_ij_)^2^, with e_ij_ = x_ij_ − Σ_k=1_^p^g_ik_f_kj_ and u_ij_ denoting the analytical uncertainty for species j in sample i [89].

PMF analysis requires two input files: a concentration file and an uncertainty file. The uncertainty file is generally calculated from the method detection limit (MDL) and the error fraction.

When c ≤ MDL, u_ij_ = (5/6) × MDL [89].

When c > MDL, u_ij_ = $\sqrt{\left[{\left(ErrorFract\;ion\times concentrat\;ion\right)}^{2}+{\left(0.5\times MDL\right)}^{2}\right]}$, where ef is the error fraction [89].

Here, c is the measured concentration, MDL is the method detection limit, and ef is the analytical error fraction. Uncertainty conventions must be matched to the PMF 5.0software version and quality control protocol used in each study.

These two sets of uncertainty calculation equations are extensively applied to construct uncertainty input files required for PMF source apportionment modelling. They have gained wide popularity in environmental pollution research covering river sediments, soils, atmospheric particulates and other typical environmental matrices, and serve as a classical and commonly accepted strategy to deal with measured concentrations above and below the method detection limit in practical PMF computation.

PMF is widely used and effective for analyzing sources of heavy metals in river sediments and other environmental media and for quantitatively assigning source contributions [9,46].

PMF also has limitations in practice. First, it cannot objectively determine the optimal number of factors, and researchers often need to set multiple factor numbers D and conduct repeated runs and interpretive screening.

To improve PMF accuracy and reliability, multi-method integration is recommended. PMF can be combined with PCA, machine learning, and isotope tracing for factor-number selection and source verification.

#### 4.3.3. UNMIX Model

The UNMIX model is a receptor model developed by Henry based on PCA. If the dataset does not meet model requirements, sources cannot be identified. UNMIX results are generally considered reliable when two criteria are met: the minimum coefficient of determination (Min R_2_) is greater than 0.8, and the minimum signal-to-noise ratio (Min S_ig_/Noise) is greater than 2.

C_ij_ = Σ F_jk_ S_ik_ + E

where C_ij_ is the content of species j in sample i; F_jk_ is the mass fraction of species j in source k (1, …, m); S_ik_ is the total amount of source k in sample i and represents source contribution; and E is the standard deviation of analysis.

UNMIX is not yet widely used in soil heavy metal source apportionment, but its self-modeling curve resolution transformation can help ensure result stability and reliability. Its advantages include simple operation, no need for predefined source profiles, no complex parameter adjustment, and direct calculation based on measured elemental concentrations.

However, UNMIX also has clear limitations. Its results depend strongly on selected marker elements; it cannot easily distinguish sources with similar contribution characteristics; and its accuracy decreases at large spatial scales or in geologically complex regions.

Model accuracy can be improved by optimizing sample size and data quality. In areas with strong spatial heterogeneity, a reasonable sampling design and joint verification with PMF and other models can enhance source apportionment credibility [54].

### 4.4. Isotope Ratios (e.g., Pb and Cd Isotopes)

Isotope ratio methods are based on isotopic mass conservation. Heavy metals from different sources often have distinctive isotopic fingerprints, and their isotopic compositions are relatively stable during physical, chemical, and biological processes. By extracting stable isotopes from receptors such as river sediments and comparing them with potential source samples, these methods can accurately quantify metal sources and contributions [46].

In source apportionment, studies often focus on ratios between two or more isotopes, such as Cd and Pb isotope ratios. High-precision instruments such as multi-collector inductively coupled plasma mass spectrometry (MC-ICP-MS) and thermal ionization mass spectrometry (TIMS) can measure small variations in isotope ratios and help identify pollutant sources and migration transformation processes. Relative isotope ratio deviations (δ values) are key indicators of analytical accuracy.

In the equation, E represents the metal element, R represents relative isotope abundance, and X and Y represent the mass numbers of two isotopes [54].

As an advanced tracing tool, isotope technology requires relatively few samples, involves straightforward calculation, and provides high discrimination accuracy, making it effective for distinguishing different pollution sources [46]. However, high analytical cost, complex sample pretreatment, environmental interference, overlapping isotopic signals, and isotopic fractionation during migration and transformation can alter original isotopic compositions and increase apportionment uncertainty.

## 5. Remediation Technology System for Heavy-Metal-Contaminated Sediments

Remediation of heavy-metal-contaminated sediments is a core component of aquatic ecological restoration. Current technologies can be broadly divided into in situ and ex situ treatment approaches [5].

### 5.1. In Situ Treatment Technologies

In situ treatment does not require sediment excavation and is implemented directly in contaminated areas. It causes less disturbance to the water body and is relatively convenient for engineering applications.

#### 5.1.1. Physical Treatment

In situ physical treatment mainly relies on sediment capping, which is widely applied. The method uses covering materials to isolate contaminated sediment from overlying water and reduce heavy metal migration and release. Common materials include natural functional soils, fly ash, modified materials, and synthetic capping materials. Although simple to operate, capping only isolates pollutants rather than removing them. It may cause secondary pollution if the cap is damaged, alter riverbed slope and flood storage capacity, and be eroded in high-flow areas. Limited availability of efficient capping materials restricts large-scale application [21,67,90].

#### 5.1.2. Chemical Treatment

In situ chemical treatment reduces sediment contamination by adding chemical reagents that immobilize or transform heavy metals. It mainly includes redox treatment and passivation. Redox approaches add oxidants such as calcium nitrate, potassium permanganate, and hydrogen peroxide to remove or transform metals through oxidation-reduction reactions [91,92].

#### 5.1.3. Bioremediation

Bioremediation uses microbial, plant, and animal metabolism to degrade, transform, or immobilize metals into less harmful forms. Compared with physical and chemical technologies, it has broad applicability, low cost, and limited environmental disturbance. Common approaches include microbial remediation and phytoremediation [90,93,94].

### 5.2. Ex Situ Treatment Technologies

**Figure 3:**
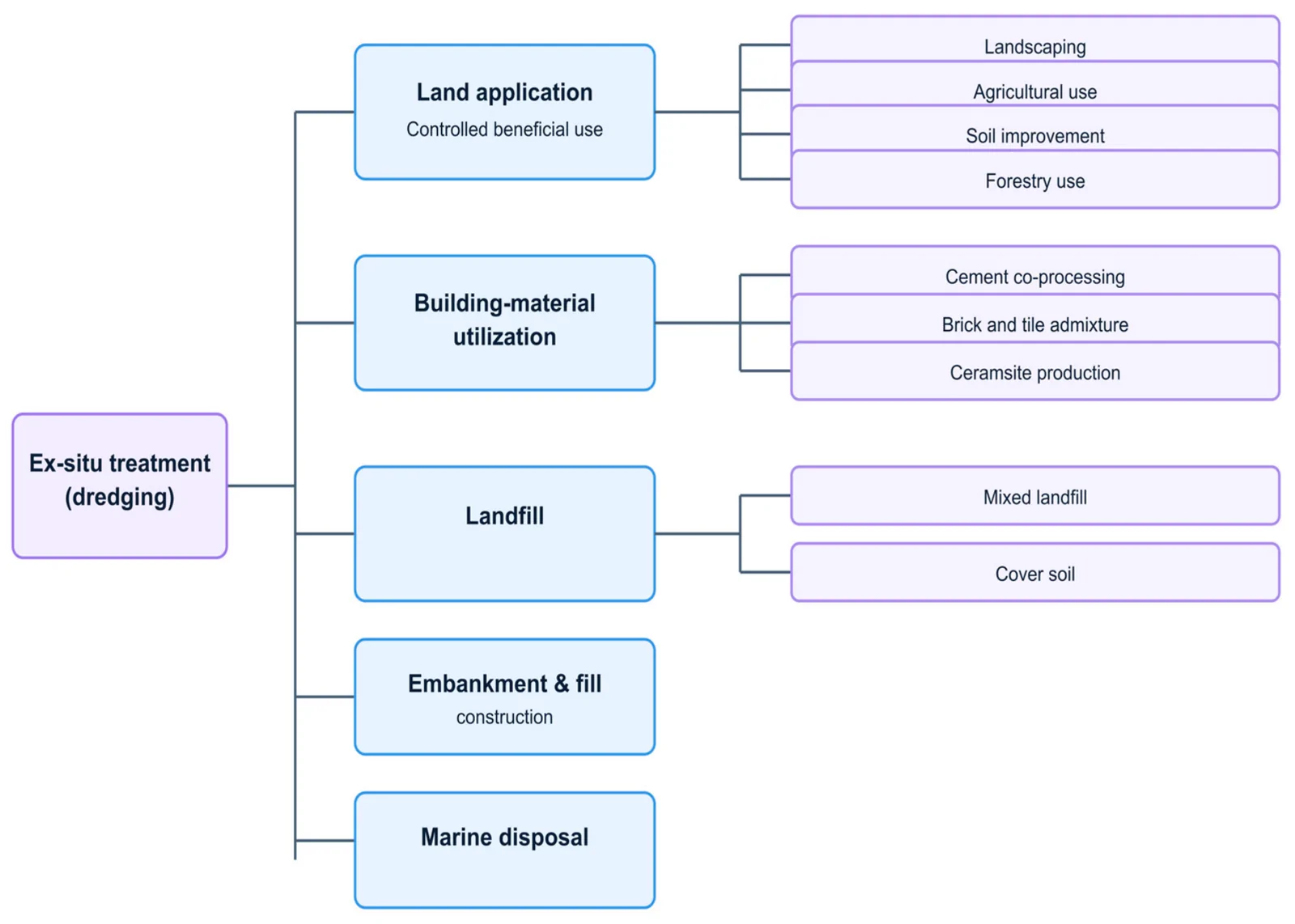
Ex situ treatment and resource utilization of dredged sediments [90].

Ex situ treatment requires excavation of contaminated sediments and transport to designated areas for centralized treatment. It is suitable for heavily contaminated sediments or situations where in situ remediation is difficult. Main pathways include land application, building material utilization, landfill, embankment and fill construction, and marine disposal [62,65,66,90]. The main ex situ treatment and resource-utilization pathways are summarized in Figure 3.

#### 5.2.1. Land Application

Sediments are rich in nutrients such as nitrogen, phosphorus, and potassium and can be used as land resources if pollutant concentrations are strictly controlled. Main applications include landscaping, agricultural use, soil improvement, and forestry. Because China lacks a specific standard for land application of dredged sediments, practice should refer to existing standards and consider application sites and soil background values to prevent secondary pollution [36,90,95].

#### 5.2.2. Building Material Utilization

River sediments contain abundant inorganic matter and can serve as alternative raw materials for bricks, cement, ceramsite, and other construction materials. This can reduce land occupation by sediment disposal and minimize clay extraction from farmland, providing environmental and social benefits. Major pathways include cement kiln co-processing, blended cement production, and co-production of ceramsite [21,90].

#### 5.2.3. Landfill

Dredged sediments can be co-landfilled with municipal solid waste, provided their quality meets standards such as GB/T 23485-2009 and GB 16889-2008 [67,90].

#### 5.2.4. Embankment and Fill Construction

After solidification, sediments can be used as fill materials for earthworks and road construction. Compared with ordinary soil, solidified sediments may show limited consolidation settlement, higher strength, and lower permeability, meeting requirements for embankment and road construction [90,96].

#### 5.2.5. Marine Disposal

Marine disposal is applicable in some coastal cities, where dredged sediments are transported to designated marine dumping areas under marine management authorities. It should comply with standards such as GB 30980-2014 [97]. With stricter marine environmental protection requirements and saturation of nearshore dumping areas, some coastal regions in China have prohibited marine sediment disposal [45,74,90].

**Figure 4:**
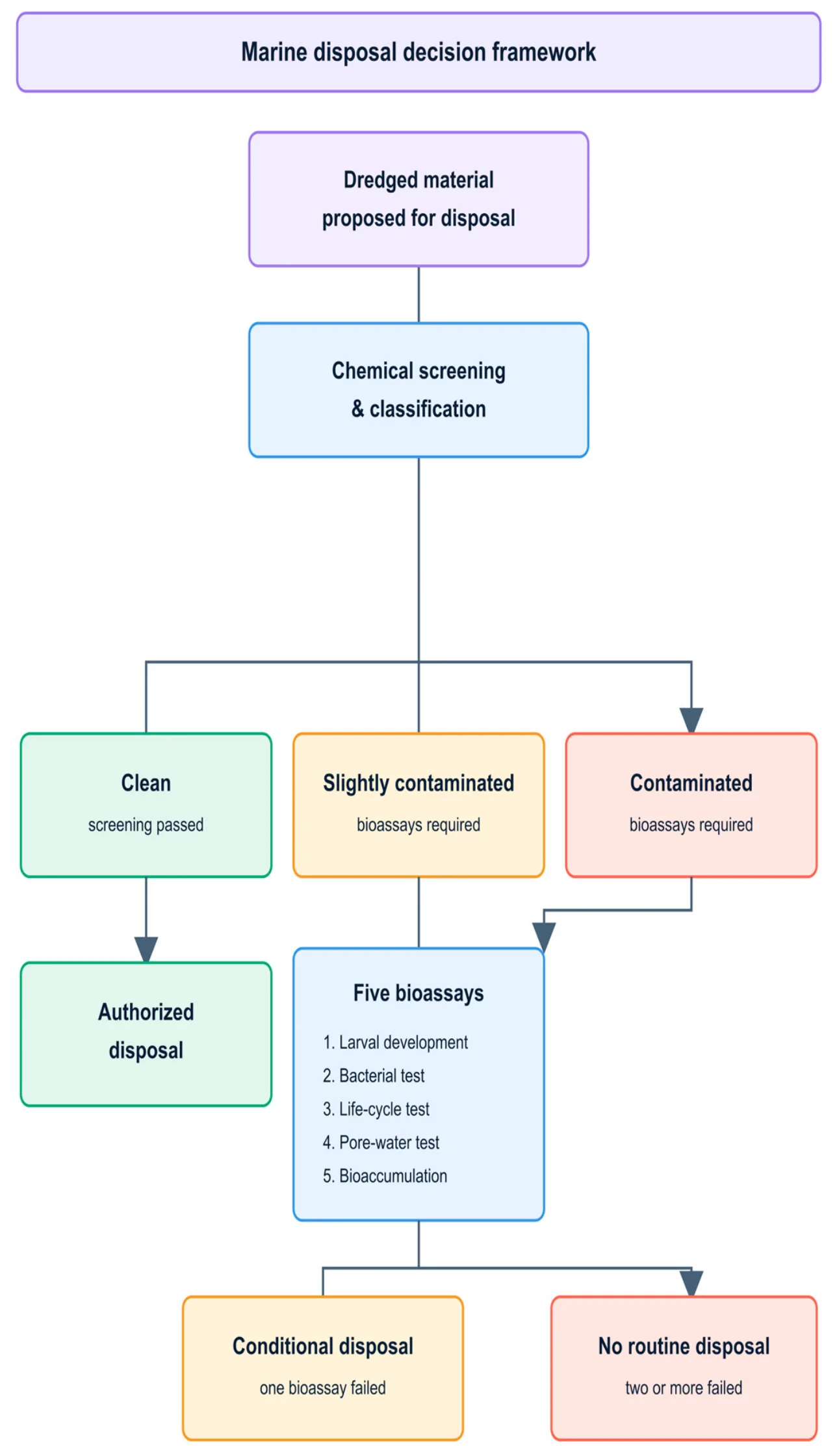
Decision framework for marine disposal of dredged material using a five-bioassay battery. The five tests cover water-phase larval development, water-phase crustacean/fish survival, whole-sediment effects on filter feeders, whole-sediment effects on deposit feeders or burrowers, and benthic bioaccumulation. Pass/fail thresholds and permit outcomes must follow the competent authority [97].

The decision framework in Figure 4 uses a five-bioassay battery: (1) a water-phase larval development test using bivalve or echinoderm larvae; (2) a water-phase survival test using a crustacean or fish; (3) a whole-sediment toxicity test using a filter-feeding benthic organism; (4) a whole-sediment toxicity test using a deposit-feeding or burrowing organism; and (5) a bioaccumulation test using a filter- or deposit-feeding benthic organism. Each assay should be compared with an appropriate control or reference sediment. A toxicity assay passes when the authority-specified endpoint does not show an unacceptable adverse effect, whereas the bioaccumulation assay passes when tissue residues remain below the applicable action criterion. The decision branches distinguish all five bioassays passed, one bioassay failed, and two or more bioassays failed. The Chinese technical procedure formally groups biological evidence into water-phase toxicity, solid-phase toxicity, and bioaccumulation; consequently, the exact test species, endpoints, effect thresholds, and permit consequences must follow the competent authority for the project jurisdiction [97].

## 6. Conclusions and Perspectives

### 6.1. Main Conclusions

River sediments are both important reservoirs of heavy metals and potential secondary sources under environmental disturbance. This review uses a global methodological framework while placing particular emphasis on Chinese river basins, where industrialization, urbanization, agricultural intensification, mining, and hydrological engineering have produced diverse contamination and risk patterns. The assessment and source apportionment approaches are transferable, but background values, toxicity thresholds, and source interpretations require regional calibration [35,39].

Research has generally progressed through three stages: early studies focused on total concentration monitoring and pollution classification; intermediate studies introduced operationally defined fractionation and ecological risk assessment with emphasis on bioavailability; and recent studies have shifted toward refined source apportionment integrating multivariate statistics, receptor models, isotope tracing, machine learning, and spatial analysis [5,35].

A relatively complete methodological system has now been established. The investigation includes surface and core sampling, ICP-MS-based total concentration determination, and BCR sequential extraction fractionation. Assessment commonly uses Igeo, EF, PLI, RI, RAC, and SQGs. Source apportionment combines PCA, CA, APCS-MLR, PMF, UNMIX, isotopes, and machine learning for qualitative and quantitative identification [5,35,39,62,94].

Pollution characteristics vary markedly among watersheds. The China-focused evidence base covers the Fenhe River, Liujiang River, Tuojiang River, Suzhou Creek, Yanhe River of the Yellow River, lower Yangtze River, Hainan rivers, and Pearl River Estuary, confirming that cascade reservoir retention, estuarine hydrodynamics, seasonal variation, and land use differences influence metal enrichment, operationally defined fractionation, and risk levels [1,11,18,26,35,43,55,65,98,99].

Overall, investigation, assessment, source apportionment, and remediation management of sediment heavy metals have formed a mature framework and provide important support for watershed governance. However, compound pollution migration and transformation, background value differences, model uncertainty, insufficient monitoring in small and medium-sized basins, and remediation costs still constrain management applications. Future work should improve standards and strengthen risk-oriented zoned management [100,101,102].

### 6.2. Current Limitations

Despite substantial progress, several problems remain, including inconsistent evaluation benchmarks, unclear fraction redistribution mechanisms, insufficient understanding of combined pollution effects, and inadequate verification of long-term remediation stability. Future research should establish region-specific assessment benchmarks, deepen studies on heavy metal-microplastic interactions, develop cost-effective and environmentally friendly in situ remediation technologies, and build decision support systems for zoned management and remediation based on risk assessment [14,16,17,19,20,45,74].

Figure 2, Figure 3 and Figure 4 were prepared as scalable vector graphics (SVG) using a code-based vector-graphics workflow and were exported as high-resolution PNG files using Sharp v0.35.3 with libvips v8.18.3 and librsvg v2.62.3, run in Node.js v24.19.0. All text, boxes, connectors, arrowheads, and color fills were generated as editable vector elements to ensure legibility and consistent rendering at publication size.

## Figures and Tables

**Table 1:** Summary of qualitative and semi quantitative models for pollution assessment.

Stage	Core Research Content	Representative Technical Methods	Main Outputs/Objectives	Summary/Implications
Early stage	Total heavy metal monitoring; qualitative description of pollution levels	Geoaccumulation index (Igeo); pollution load index (PLI)	Establish a preliminary pollution-level assessment framework	Foundational research stage
Intermediate stage	Heavy metal speciation; bioavailability	BCR sequential extraction	Clarify speciation and bioavailability	Mechanistic understanding stage
Deepening stage	Source apportionment; risk assessment; remediation technology development	Multivariate statistics and receptor models; multi-type remediation technologies	Develop a complete research technology system; achieve source identification	System improvement stage
Current limitations	Microscale mechanisms; remediation synergy; insufficient long-term risk prediction	Lack of unified investigation and evaluation criteria; insufficient interdisciplinary integration	A whole-watershed systematic management and control framework has not yet been formed	Area requiring further work
Future research directions	Compound pollution mechanisms; high-resolution source apportionment; engineering remediation; early warning system construction	Machine learning and isotope tracing; interdisciplinary integration	Support watershed aquatic ecosystem protection and water quality improvement	Priority research direction

## Data Availability

No new data were created or analyzed in this study. Data sharing is not applicable to this article.
